# Evaluation of imaging techniques for early detection of intrathoracic cancers in symptomatic patients in primary care: a systematic review

**DOI:** 10.1136/bmjopen-2024-091435

**Published:** 2025-08-16

**Authors:** Bogdan Grigore, Jaime L Peters, Wasim Hamad, Natalia Calanzani, Lauren Asare, Fiona M Walter, Richard Neal

**Affiliations:** 1 Exeter Test Group, Department of Health and Community Sciences, University of Exeter, Exeter, UK; 2 Institute for Health and Primary Care, Queen Mary University of London, London, UK; 3 Academic Primary Care, University of Aberdeen Institute of Applied Health Sciences, Aberdeen, UK; 4 Department of Public Health and Primary Care, University of Cambridge, Cambridge, UK; 5 University of Exeter, Exeter, UK; 6 Wolfson Institute of Population Health, Queen Mary University of London, London, UK

**Keywords:** Diagnostic Imaging, Primary Care, Lung Diseases

## Abstract

**Abstract:**

**Objectives:**

Intrathoracic cancers, such as lung cancer, mesothelioma and thymoma, represent diagnostic challenges in primary care. We aimed to summarise evidence on the performance of imaging techniques that could aid the detection of intrathoracic cancers in low prevalence settings.

**Design:**

Systematic review and quality appraisal using Quality Assessment of Diagnostic Accuracy Studies-2 and Grading of Recommendations Assessment, Development and Evaluation.

**Data sources:**

MEDLINE, Embase and Web of Science were searched with a predesigned search strategy for articles from January 2000 to January 2024.

**Eligibility criteria:**

We included studies relevant for primary care, where participants were suspected of having intrathoracic cancer and reported on at least one diagnostic performance measure. We excluded studies where the cancer diagnosis was already established. Data extraction and synthesis screening were conducted independently by two reviewers. Data extraction and quality appraisal were conducted by one reviewer and checked by a second reviewer.

**Results:**

Out of 30 539 records identified by the database searches, 13 studies were included. There was heterogeneity in the types of cancers, populations included and reported diagnosis pathways for suspected cancers. Imaging modalities investigated included chest X-ray (three studies), computer tomography (CT, six studies), magnetic resonance imaging (two studies), positron emission tomography CT (two studies), ultrasound (two studies) and scintigraphy (one study). Chest X-ray sensitivity reported for lung cancer ranged from 33.3% to 75.9%, with specificity ranging from 83.2% to 95.5%. For CT, reported sensitivity varied from 58% for pleural malignancy to 100% for lung cancer. One study investigating an artificial intelligence tool to detect lung cancer found poor detection performance in a real-world patient cohort.

**Conclusions:**

We found a limited number of studies reporting on the diagnostic performance of usual imaging techniques when used in unselected primary care settings for the diagnosis of intrathoracic cancer in symptomatic patients. There is a need for more studies evaluating such techniques in the general population presenting in primary care, where the prevalence is relatively low. A better understanding of the performance could lead to better detection strategies for intrathoracic cancers in primary care. Intrathoracic cancers, such as lung cancer, mesothelioma and thymoma, represent diagnostic challenges in primary care. We aimed to summarise evidence on the performance of imaging techniques that could aid the detection of intrathoracic cancers in low prevalence settings.

**STRENGTHS AND LIMITATIONS OF THIS STUDY**

- The systematic review used a comprehensive search of the literature for studies reporting on diagnostic performance of imaging in primary care patients.
- Studies reporting on impact of diagnostic imaging in primary care, as well as studies reporting the use of artificial intelligence in the primary care setting, were also sought.
- We used Quality Assessment of Diagnostic Accuracy Studies-2 to evaluate the risk of bias and the Grading of Recommendation, Assessment, Development and Evaluation approach to evaluate the strength and quality of the evidence.
- Very few studies focused on primary care symptomatic patients, most of them included small samples and reported limited accuracy data.

## Background

 Intrathoracic cancers including lung cancer, mesothelioma and thymoma remain a diagnostic challenge as they have similar presentation to other conditions. They can be ‘easily missed’ in primary care and are often diagnosed at a late stage.^1^ However, many patients experience symptoms and may have detectable imaging findings prior to their diagnosis. Thereby, creating a time window in which imaging may facilitate detection at earlier stages, leading to less invasive treatments and improving survival.^2^

Imaging is fundamental to the investigation of suspected intrathoracic malignancies. During their clinical journey, patients with suspected intrathoracic cancer may undergo a number of imaging modalities. Chest X-ray (CXR) is usually the first diagnostic imaging in suspected intrathoracic cancer. The England and Wales National Institute for Health and Care Excellence (NICE) guidelines^3^ recommend a CXR within 2 weeks when a lung or pleural malignancy is suspected (if the patient is over 40 years old and presents with two or more suggestive symptoms).^4^ While CXR is a relatively accessible investigation in primary care, existing evidence suggests that its sensitivity for lung cancer is limited,^5^ especially for early stages,^6^ with recommendations for general practitioners (GPs) to seek further investigations in high-risk patients with negative CXR.^5 7^

The second most used imaging technique for diagnosing intrathoracic cancers is CT.^8^ Patients with suspected intrathoracic cancer are often offered contrast-enhanced CT to further the diagnosis or to provide information on the cancer staging.^4^ CT is used as part of different investigative approaches, such as screening,^9^ and biopsy guidance,^10^ and cancer staging.^3 7^

Positron emission tomography CT (PET-CT) is an imaging technique that employs radioactive contrast substances to generate gamma rays that are captured as images.^8^ Many guidelines^4 11^ recommend that all patients with potentially curable cancer have an initial evaluation with PET-CT to evaluate for metastasis and establish cancer stage.

While not recommended for the investigation of primary lung cancer,^7^ MRI is useful in identifying rarer forms of cancer, for example, pleural mesotheliomas^12^ alongside CT.^8^ Ultrasound (US) techniques are not usually used for investigating suspected intrathoracic cancers in most settings; however, they can provide valuable information on the condition of the chest wall, pleurae and lymph nodes.^8^

Scintigraphy is an imaging technique where gamma radiation is captured (as in PET-CT);^13^ however, it lacks three-dimensional information. Scintigraphy is a less used method due in part to the greater accessibility of other imaging techniques.^14^ Recent studies suggest that scintigraphy with somatostatin analogues may help differentiate between malignant and benign tumours.^15 16^

There is increasing evidence on the performance of these and other imaging techniques; however, most of this evidence is concerned with populations in secondary and tertiary care, where cancer prevalence is higher than that of the general population. There has been, however, less research into the use of imaging in primary care for cancer detection, where cancer prevalence is much lower. The distinction is important as these techniques may not perform as well in low prevalence populations due to spectrum bias effect;^17^ they are likely to have a lower sensitivity and higher specificity in primary care.

General practitioners play a crucial role in investigating or referring symptomatic patients along appropriate diagnostic pathways. With increasing pressure on healthcare systems, there is a need to avoid, where feasible, referring symptomatic patients where a cancer diagnosis is unlikely. Currently, many countries, including the UK, are implementing CT screening of high-risk asymptomatic populations. However, this will only account for the diagnosis of a small percentage of all intrathoracic cancers, with the majority continuing to be diagnosed through a symptomatic route. Furthermore, accuracy evidence from screening studies, while largely available, may not pertain to symptomatic patients as the underlying risk of cancer is different.

The current study was conducted within the CanTest framework,^18^ an initiative with the aim of improving detection of cancer in primary care, in view of more effective implementation into practice.^18^ For this systematic review, we aimed to identify and synthesise studies reporting on the performance, feasibility and impact of imaging techniques for the detection/diagnosis of intrathoracic (primary lung/bronchus/trachea, mesothelioma and thymoma) cancers, which have potential relevance to primary care.

## Methods

This systematic review was developed in accordance with the Preferred Reporting Items for Systematic Reviews and Meta-Analysis (PRISMA) guidelines^19^ and was registered in the PROSPERO database (CRD42023407698).

### Search strategy

Scoping database searches suggested that a high number of papers would need to be screened, due to many potentially relevant reports being indexed in non-related categories (eg, papers that may be applicable to primary care but do not mention this setting). Our search strategy was designed to provide a reasonable balance between sensitivity and number of papers to be screened. In order to achieve this, it was conducted in two steps. In the first step, we conducted a database search for studies reporting on diagnostic performance of imaging techniques. We searched MEDLINE, Embase and Web of Science with a predesigned search strategy (using free text and database-specific subject headings, see online supplemental material, appendix 1). The search was restricted to human studies published after 1 January 2000, in any language. Potentially relevant papers identiﬁed from other sources (such as newsletters and discussions with colleagues) were also considered. In the second step, we also conducted a search of references of included papers, as well as papers citing these papers. In this step, we also included any available studies reporting on implementation (such as feasibility and acceptability) and impact (such as clinical utility and/or cost-effectiveness).

Inclusion and exclusion criteria are described in table 1.

**Table 1:** Inclusion and exclusion criteria

	Inclusion	Exclusion
Population	Adults with a suspicion of primary intrathoracic cancers (lung/bronchial/tracheal cancer, mesothelioma and thymoma), but where cancer diagnosis is not established at recruitment.	Adults with established/known cancer diagnosis at recruitment, or in participants with intrathoracic metastases or history of intrathoracic cancers Asymptomatic adults with average risk (ie, screening) Studies in vitro or on animals
Intervention	Imaging techniques that would be suitable for use to aid diagnosis of intrathoracic cancer in primary care.	Biopsy procedures
Comparators	Individuals who do not have a diagnosis of cancer—non-malignant conditions such as benign nodules, COPD or others are eligible comparators	–
Outcome	Diagnostic accuracy/performance of imaging techniques including sensitivity, speciﬁcity, positive predictive value, negative predictive value and area under the curve. Where reported, data on the implementation (eg, feasibility and acceptability) of imaging in this context, as well as the impact of imaging, such as clinical utility (eg, reduced time to diagnosis or treatment) and/or cost-effectiveness	
Publication type	Quantitative studies reporting results from primary data (RCTs and observational studies) published in peer-reviewed journals	Abstracts presented at conferences/meetings without an accompanying full-text publication; qualitative studies; reviews, systematic reviews and overviews

COPD, chronic obstructive pulmonary disease; RCTs, randomised controlled trials.

### Study screening

Study screening adhered to PRISMA flowchart.^19^

Studies identiﬁed from searches were screened for relevance based on title and abstract. Screening was conducted independently by two reviewers using Rayyan.^20^ A pilot exercise took place between all reviewers for a number of the hits, to ensure inter-reviewer consistency.

Discussion between reviewers or with a third reviewer resolved any disagreement. Full text of studies included at the title and abstract screening stage was then screened independently by two reviewers.

### Data extraction

A data extraction form was developed and piloted to ensure consistency in extraction between studies and reviewers. Three included papers were used in the pilot phase and the data extraction form was amended as necessary. The process was iterative, with data being extracted independently by each reviewer, followed by discussion as to the clarity and completeness of the data extraction form and individual questions. Data extraction was then carried out independently by one reviewer and checked by a second. Disagreements and differences in data extraction were resolved by discussion and consensus, where necessary. While the experience and area of expertise of the reviewers in the team varied, two experienced systematic reviewers (BG, JP) ensured the process was methodologically rigorous.

We extracted information on: study characteristics (publication year, country of population of interest, recruitment setting, study aims and design); populations (sample size, age, sex, tumour staging for people with cancer and health status for people without cancer); imaging technique (including technical specifications/characteristics) and summary measures of diagnostic performance (sensitivity, specificity, positive predictive value, negative predictive value, area under the curve). Where reported, we also extracted data on the implementation (eg, feasibility and acceptability) of imaging in this context, as well as the impact of imaging, such as clinical utility and/or cost-effectiveness.

### Quality assessment

To assess the risk of bias (RoB), we used the Quality Assessment of Diagnostic Accuracy Studies-2 (QUADAS-2) tool^21^; for comparative accuracy, we also used Quality Assessment of Diagnostic Accuracy Studies-Comparative (QUADAS-C).^22^ QUADAS-2 evaluates the RoB based on four domains (patient selection, index test, reference standard, flow and timing). The first three domains are also assessed in terms of applicability. QUADAS-C is an extension of QUADAS-2, based on the same four domains, but with additional questions relevant to test comparisons.^22^ For studies reporting outcomes related to implementation or impact, we planned to use the Cochrane Collaboration’s ‘Risk of Bias’ Tool^23^ or the ROBINS-I (Risk of Bias in Non-randomised Studies-Interventions) tool.^24^ For quality appraisal, two reviewers assessed the included papers independently, after piloting the approach on the first three papers; disagreements were discussed and consensus was reached. No automation tool was used in the process.

### Data synthesis

Extracted data were presented narratively and in summary tables by imaging technique. All relevant outcome measures from the included studies were reported. Where available, implementation and impact outcomes (including feasibility, acceptability and cost-effectiveness) were also synthesised using narrative synthesis. Diagnostic results were mainly summarised in terms of sensitivity, specificity, positive predictive value (PPV) and negative predictive value (NPV). Where these data were not available, but contingency tables could be reconstituted, we reported data from these tables instead. Key critical appraisal aspects were also included in the narrative synthesis.

Heterogeneity across included studies in imaging techniques, populations, study designs and outcome measures precluded meta-analysis.

### Quality of GRADE evidence

To evaluate the quality of evidence, we applied the Grading of Recommendations Assessment, Development and Evaluation (GRADE) methodology^25^ adapted for tests.^26^ Two researchers (BG and JP) independently conducted the assessment. Disagreements were discussed and, when necessary, a third researcher (RN) helped the reach of a consensus. The final quality of evidence was categorised into one of three levels—high, moderate, low and very low.

### Patient and Public Involvement (PPIE)

A patient and public involvement and engagement (PPIE) panel was established and consulted throughout the project. In the development of the proposal for funding, and in the development of the protocol, the PPIE panel was very supportive of the need for the work and the approaches that we were taking. Two specific issues were raised. First, the suggestion that we should measure test performance in different ethnic groups led to the potential inclusion of subgroup analysis if the data permitted. The second related to measuring harms as a consequence of testing, as well as issues relating to the process and acceptance to testing (including issues such as waiting times, the need to travel, the need for ‘preparation’, the stress and anxiety working up to the test). We sought to report on any of these aspects in the included studies.

At the end of the study, we held a PPIE workshop where we discussed the meaning of the results. The PPIE members were supportive of our findings and conclusions, but again raised issues (beyond the scope of the review) about the process and acceptability of testing.

## Results

### Study screening

**Figure 1:**
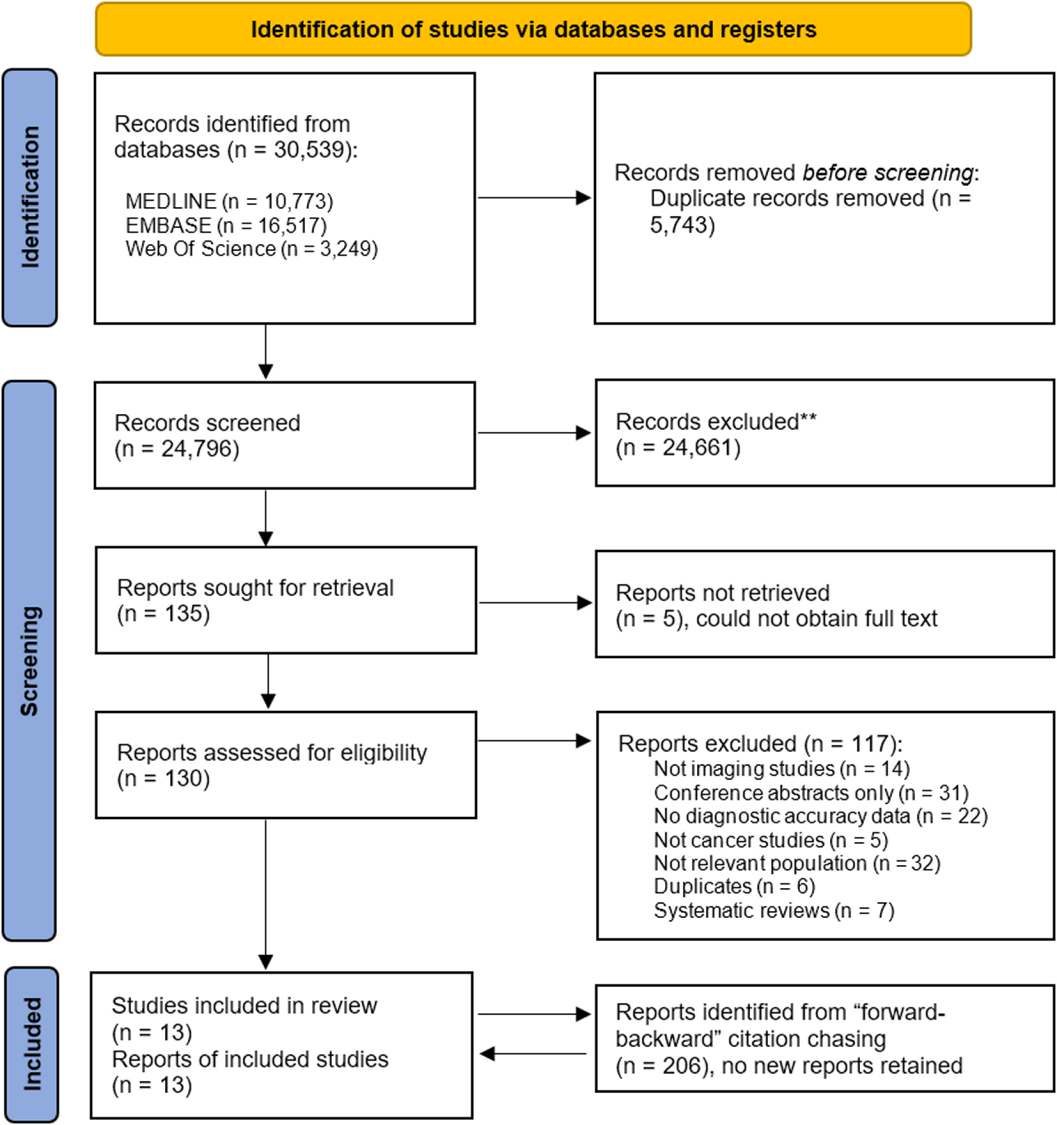
PRISMA diagram of screened papers. PRISMA, Preferred Reporting Items for Systematic Reviews and Meta-Analysis.

Database searches were run in January 2023 and resulted in 21 567 potentially eligible studies. A search update was conducted in January 2024, which resulted in 3399 additional papers. After deduplication, 24 796 studies were identified for title and abstract screening, and 130 papers were assessed in full-text screening. The most likely reasons for exclusion at full-text level were the population not being compatible with our inclusion criteria, the non-availability of full text and the lack of reported accuracy data. 13 papers were included in our review (see figure 1).

An additional search was carried out to identify references and citations of the included papers. 206 papers were identified. After title and abstract screening, two papers were screened in full. No additional papers were included following this step.

Of the 13 studies included, five were conducted in the UK,^27^,^31^ four in China,^32^,^35^ two in Germany^36 37^ and one each in France^38^ and Italy.^39^ Three studies reported on CXR,^27^,^29^ six on CT,^30^,^3236^ two on MRI,^32 37^ two on PET-CT,^33 35^ two on ultrasound^34 39^ and one on scintigraphy.^33^ Reference standards used varied across the reports from biopsy and histopathologic analysis,^30^,^3537 38^ clinical reports^28 29 39^ to registries^27 36^ and death records.^36^

Study sample size varied greatly, from 28^37^ to 8996.^28^ Most participants were selected based on the symptomatic/clinical suspicion of lung cancer, with the exception of two studies, where asymptomatic, but high-risk (due to asbestos exposure) patients were included,^36 38^ and one study where participants presented with respiratory symptoms, but not specific to cancer.^39^

**Figure 2:**
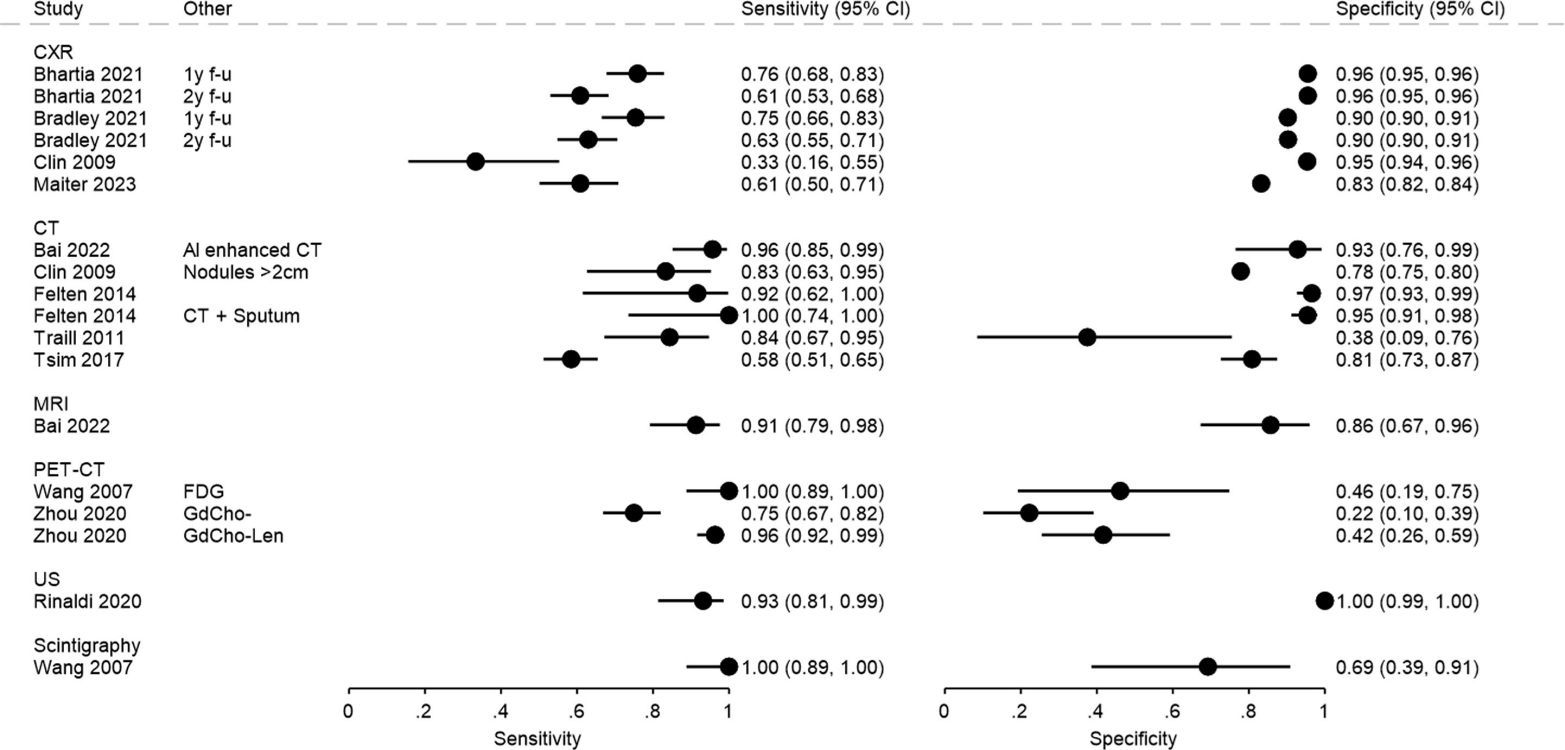
Accuracy data extracted from the included papers.

A summary of included studies is presented below, starting with those including symptomatic patients, followed by studies including asymptomatic patients (see online supplemental material, appendix 2 for all extracted data). Summaries of the risk of bias are presented in table 2, with estimates of accuracy shown in figure 2 (and online supplemental material, appendix 3). The reporting of accuracy data was heterogeneous. Where possible, 2×2 contingency tables were extracted or reconstructed by the review authors to report estimates of sensitivity, specificity, PPV and NPV. The majority of the studies did not report area under receiver operator characteristics (AUROC). Only Yang *et al*^34^ report this data explicitly, while Rinaldi *et al* report AUROC for all diagnoses (of which cancer diagnoses represented only 11%) and Zhou reports a graph indicating the relationship between specificity and sensitivity, without any further detail.

**Table 2:** Critical appraisal of studies according to QUADAS-2

Study	Risk of bias				Applicability concerns		
	Patient selection	Index test	Reference standard	Flow and timing	Patient selection	Index test	Reference standard
CXR							
Bhartia *et al* 2021^27^	☺	☺	☺	?	☺	☺	☺
Bradley *et al* 2021^28^	☺	☺	?	?	☺	☺	?
Maiter *et al* 2023^29^	☺	☺	☺	☺	☺	☺	☺
Clin *et al* 2009^38^	?	☺	?	?	?	☺	?
CT							
Bai *et al* 2022^32^	?	?	?	☺	?	?	?
Felten *et al* 2014^36^	?	☺	☹	☺	?	☺	☺
Traill *et al* 2001^30^	?	☺	☺	☺	☺	☺	☺
Tsim *et al* 2017^31^	☺	☺	?	☺	☺	☺	☹
MRI							
Heye *et al* 2012^37^	?	☺	☺	☺	☹	☺	☹
PET-CT							
Yang *et al* 2022^34^	☺	?	☺	☺	☺	☹	☹
Zhou *et al* 2020^35^	?	☹	☺	☺	?	☹	☺
Ultrasound							
Rinaldi *et al* 2020^39^	☺	?	?	?	☹	☹	?
Scintigraphy							
Wang *et al* 2007^33^	?	☺	?	?	?	☺	?

☺: low risk; ☹: high risk; ?: unclear risk.

CXR, chest X-ray; PET-CT, positron emission tomography CT; QUADAS-2, Quality Assessment of Diagnostic Accuracy Studies-2.

### Studies including symptomatic patients

#### Chest X-ray studies

Bhartia *et al*^27^ reported on a prospective study where patients with symptoms deemed eligible were able to self-refer for a CXR. 8948 examinations of patients aged 50 years or more were included. The reference standard was diagnosis of any primary intrathoracic cancer or specifically non-small cell lung cancer (NSCLC), within 1 year and 2 years from imaging. There were no significant differences between the sensitivity and specificity of detection for all intrathoracic cancers and NSCLC. This study was appraised as having low risk of bias, with minimal concerns in all appraised domains.

Bradley *et al*^28^ used a cohort from the same study as Bhartia *et al*^27^ and explored accuracy of CXR in conjunction with relevant, but non-specific symptoms (eg, cough, haemoptysis, breathlessness, chest pain, weight loss, change in voice) to assist diagnosis of (any) lung cancer. The authors reported a sensitivity of CXR to detect lung cancer within 1 year to be 75.4% (CI 67.5% to 83.3%), and specificity 90.2% (CI 89.6% to 90.9%). These figures are not directly comparable to findings by Bhartia *et al*, as there were slight differences in the samples (8996 vs 8948 participants) due to different exclusion criteria, as well as in the outcomes (lung cancers vs all intrathoracic cancers and NSCLC only, respectively). Bradley *et al* reported PPV for presence of symptoms in case of a negative CXR and found the PPV of all symptoms for a diagnosis of lung cancer within 1 year of CXR to be <1% for all individual symptoms except for haemoptysis, which had a PPV of 2.9%. This study generally presented low risk of bias and low concerns; however, the risk of bias concerning the reference standard was deemed unclear, due to relying on the hospital database; presumably, if patients moved away, their diagnoses would no longer be recorded.

Maiter *et al*^29^ evaluated the diagnostic performance of an artificial intelligence (AI) solution for lung nodule detection (Auto Lung Nodule Detection software, ALND; Samsung Electronics, Suwon, South Korea) applied retrospectively to a set of CXRs acquired on the request of GPs. 5722 CXR were included in the study and the detection performance of the AI software was assessed against the cancer diagnosis from a multidisciplinary team. The AI software underperformed against the multidisciplinary team with a PPV of 5.6% (95% CI 4.8% to 6.6%). According to the authors, this may be due to the AI training and validation using “insufficiently representative and generalisable datasets”^29^ (enriched datasets from secondary centres in Korea, Germany and the USA), reflecting a higher prevalence than in primary care. The study was assessed as having low risk of bias and low applicability concerns.

#### CT studies

The study conducted by Traill *et al*^30^ aimed to determine the sensitivity and specificity of contrast-enhanced CT (CE-CT) for the detection of pleural malignancy in patients referred for the diagnosis of a suspected malignant pleural effusion (MPE). 40 consecutive patients participated in the study, of whom 32 had a malignant pleural effusion. Overall, based on figures reported by the authors, sensitivity for CE-CT was 84.4% for detecting MPE, and the specificity was 62.5%. Risk of bias was assessed as low for the index test, reference standard and patient flow, but high for patient selection, due to limited information reported on this aspect. The small sample reported and the high prevalence of malignancy also raised concerns.

Tsim *et al*^31^ conducted a retrospective analysis of 315 consecutive patients who underwent CE-CT for suspected pleural malignancy. The reference standard was a mix of cytological or histological confirmation, or follow-up of disease progression for at least 6 months. The authors reported CE-CT to have an overall sensitivity of 58% and a specificity of 80% for detecting pleural malignancy; however, only 59 (19%) of the 195 cases detected represented primary mesothelioma, with the rest indicating secondary malignancies. Due to this, the study raised several concerns for high risk of bias regarding the sample included. Another concern was raised by the study using multiple reference standards, varying from histopathological confirmation to consensus over progression in the multidisciplinary team.

#### CT and MRI studies

Bai *et al*^32^ explored the diagnostic effect of high-resolution reorganisation of medical images based on deep convolutional neural networks (CNN) in lung cancer diagnostics. The study included 74 patients highly suspected of lung cancer based on symptoms (cough, vocal cord tearing, expectoration and blood in sputum). Patients were examined by CT and MRI, and images from both were enhanced with a deep CNN algorithm. All patients underwent biopsy for diagnostic confirmation. CT-guided diagnostic accuracy was calculated as 94.6%, while MRI-guided diagnosis was 89.2% (see figure 2). Authors concluded that both techniques were highly accurate, although they performed differently from one another: CT had shorter procedure time, while it involved radiation, and MRI had longer time, while not involving any radiation. The study was appraised as having unclear risk of bias for the patient selection, index test and interpretation of the reference standard, due to limited information reported on how the patient sample was obtained and the context of index test and reference standard interpretation (eg, timing, blinding). There were also some concerns regarding the applicability of the index test, given that only patients highly suspected of cancer were included in the study.

#### MRI studies

Heye *et al*^37^ reported on a study that assessed the accuracy of lesion detection by MRI against CT as reference standard. The prospective study included 28 patients with suspected thoracic malignancy and clinical indication of chest diagnostic imaging. All patients underwent MRI and CT. The authors found MRI to be concordant with CT in detecting primary thoracic malignancies, as well as ruling out pulmonary lesions; however, MRI detected only 28%–36% (depending on the sequence used) of the ≥5 mm nodules detected by CT (49%–59%). The study had low risk of bias on the conduct of index test and patient flow, with unclear level of risk of bias for patient selection (due to limited information reported on this step) as well as reference standard (with both MRI and CT conducted by the same observers). There were significant applicability concerns regarding the sample used (26 of the 28 participants had malignancies) as well as the use of CT as reference standard, despite having access to pathophysiological information (ie, which could have offered an arguably better reference standard).

#### PET-CT studies

In the study by Zhou *et al*,^35^ 172 patients with suspected lung cancer underwent PET-CT with Gd_2_O_3_-doped carbon-11-choline-lenvatinib (GdCho-Len-PET) nanoparticles contrast agent in order to evaluate its diagnostic value. The authors found that GdCho-Len-PET presented with higher sensitivity (96.3%) and specificity (42.7%) compared with GdCho-PET contrast agent (75% and 22.2% respectively). There were some concerns related to the index test used, both in terms of risk of bias and applicability, due to very early use of PET-CT in the diagnostic pathway.

#### Ultrasound studies

In their 2020 study, Rinaldi *et al*^39^ explored the utility of role of lung ultrasound (LUS) in the clinical diagnostic algorithm of respiratory diseases. 509 patients admitted for respiratory symptoms were prospectively included and evaluated with LUS. Authors found that LUS findings were highly concordant with the reference standard (41/44 cases were found positive in LUS); however, this was mostly due to the frequent finding of indirect signs such as pleural effusion or thickening, large areas of atelectasis and secondary peripheral lesions. Due to the limited reported information specifically on cancer diagnosis, the risk of bias could not be determined on most domains. There were significant concerns regarding the population included in the study, as it included patients without suspected intrathoracic cancer.

Yang *et al*^34^ conducted a study exploring the diagnostic capabilities of high-frequency B-mode US and contrast-enhanced US (CEUS) in terms of differentiating between benign and malignant pleural diseases in a study that enrolled 50 consecutive patients. The authors found that both B-mode US and CEUS were able to clearly distinguish between benign and malignant thickened pleurae (area under the curve 0.819 for B-mode US and 0.848 for CEUS). The study was evaluated as having a low risk of bias on patient selection, reference standard and patient flow, but unclear risk of bias on the index test. This was due to the uncertain definition of a positive result. The study was judged as high risk of bias for applicability of the index test and reference standard.

#### Scintigraphy study

Wang *et al*^33^ evaluated the clinical value of tomographic ^99m^Tc-octreotide scintigraphy in comparison with that of fluorine-18 fluorodeoxyglucose (^18^F-FDG) dual-head coincidence imaging (DHC) in a sample of 44 consecutive patients with suspected lung cancer. Diagnosis was confirmed by histopathologic findings or clinical follow-up. The authors concluded that the sensitivity (100%) and specificity (69.2%) of tomographic ^99m^Tc-octreotide scintigraphy were comparable to those of ^18^F-FDG coincidence imaging (100% and 46.2%, respectively) for the primary lesions. The risk of bias could not be determined for most of the domains due to lack of details on the reference standard, length of follow-up and reporting of blinding. There were concerns regarding applicability to a primary care setting as there was a high prevalence of cancer (31 of the 44 patients had cancer).

### Studies including asymptomatic patients

#### CXR and CT study

The study by Clin *et al*^38^ explored the performance of CXR and CT in screening for lung cancer in an asbestos-exposed population. 972 patients underwent 1230 investigations with CXR and CT to identify early lung cancer. Bronchopulmonary cancer was detected with a rate of 0.82% for CXR and 2.16% for chest CT. At a threshold of 2 mm nodules, CXR had a sensitivity of 33% and a specificity of 95%, while CT had a sensitivity of 83% and a specificity of 78%.

The study exhibited low risk of bias regarding the interpretation of the index tests (as chest radiographs were interpreted independently from the CT scans); however, the risk of bias could not be determined for the other domains, such as the reference standard (only test positive patients having received a biopsy, while data for test negative patients were obtained through follow-up) as well as the relevance of the population (a screening, high-risk population).

#### CT study

In a study by Felten *et al*,^36^ 187 high risk, asymptomatic, asbestos-exposed patients were screened for lung cancer with low-dose CT and sputum cytology; participants were followed up for more than 3 years. Reference standard was considered as confirmation biopsy where available, records in an occupational registry or death certificates. On critical appraisal, this study raised considerable concerns on the patient selection domain (with highly selected, but asymptomatic patients), as well as some concerns on the reference standard used (due to reliance on an occupational registry for some of the patient outcomes) and patient flow (due to some patients receiving a different reference standard).

#### Quality appraisal

Overall, studies varied greatly in the assessed risk of bias and applicability through QUADAS-2 (see table 2 and online supplemental material, appendix 4). The GRADE assessment downgraded a number of studies, and only Bhartia, Bradley, Maiter, Tsim and Yang were judged as high quality of evidence (see online supplemental material, appendix 5).

## Discussion

Our study employed a search strategy that resulted in a significant number of records to screen for eligibility, but only 13 studies met our inclusion criteria. All included studies were diagnostic accuracy studies. Although the search strategy was developed to identify diagnostic accuracy studies, the additional searching undertaken did not identify any relevant feasibility or impact studies. However, some of the included studies mentioned feasibility aspects. For instance, Bai *et al*^32^ noted an important trade-off of using MRI (longer scanning time, but non-radioactive) compared with CT.

The included studies covered a wide range of imaging techniques to assist the diagnosis of patients suspected of lung cancer. These were mainly on the diagnostic performance of CT, CXR and PET-CT, with a few studies also reporting on MRI, scintigraphy and ultrasound.

There was substantial heterogeneity in types of target cancers, populations included and reported pathways for the diagnosis and management of suspected cancers. In these studies, CXR sensitivity for lung cancer ranged from 33.3%^38^ to 75.9%,^27^ with specificity ranging from 83.2%^29^ to 95.5%.^27^ For CT, sensitivity varied from 58%^31^ for pleural malignancy to 100%^36^ for lung cancer (when used in combination with sputum cytology). For MRI, a sensitivity of 91.3% and a specificity of 85.7% for lung cancer were reported.^32^ Values reported for PET-CT varied for lung cancer sensitivity from 75%^35^ (using Gd_2_O_3_-doped carbon-11-choline as contrast substance) to 100%^33^ (using 18-F-fluorodeoxy glucose); from the same studies, values for specificity ranged from 22.2%^35^ to 46.2%.^33^ Rinaldi *et al*^39^ reported a sensitivity of 93.2% and a specificity of 100% for LUS in identifying lung cancer. For scintigraphy, reported sensitivity was 100% and specificity was 69.2%^33^ (using ^99m^Tc-octreotide acetate as contrast).

We identified only one report evaluating the use of an AI system in a primary care context; in this report,^29^ the particular AI solution used underperformed compared with a multidisciplinary team in the detection of cancer.^29^ This outcome highlights the broader issue of many AI imaging tools, where datasets used for training and validation do not reflect the prevalence of the general population.

### Strengths and limitations

Our review question focused on applicability in the primary care settings. For this reason, we excluded all studies where patients had been explicitly referred to secondary/tertiary care, or where the sample included both suspected and confirmed cancer cases. Due to the very large number of search records obtained, we had to focus the search strategy to a manageable output of just over 20 000 hits. However, each article was manually screened independently by two reviewers. Consequently, although unlikely, it is possible that relevant studies were missed in this review.

Our analysis focused on symptomatic populations, as symptomatic presentation within primary care remains the predominant route for identifying intrathoracic cancers in health systems with universal healthcare, particularly in the UK.^1^ Although there is a growing body of evidence on the performance of various imaging modalities for cancer screening, the fundamental differences between symptomatic and asymptomatic populations mean that this evidence is not directly transferable between the two groups, primarily because of the different performance metrics of the tests in populations with a different incidence of cancer. For this reason, we excluded the more extensive literature on screening studies involving low-risk, asymptomatic populations and confined our analysis to symptomatic detection.

The majority of the studies included cohorts of patients with high suspicion of cancer, suggesting a significantly higher prevalence of intrathoracic cancers compared with that from unselected primary care settings. Due to this, diagnostic performance data may not be generalisable to primary care settings, where many patients may present with mild symptoms.

Many of the included studies had small samples, with just half of the studies including more than 100 participants. Of these, studies by Bhartia *et al*^27^ and Bradley *et al*^28^ included a largely overlapping population, derived from the same original cohort. Due to the limited number of patients included across all studies, there is still significant uncertainty as to the performance of these imaging modalities in unselected populations. Furthermore, as the risk of bias assessment highlighted, in a number of studies, it was unclear how patients were identified, whether certain patients were excluded from the studies and whether the way the reference standard was conducted could have biased the study results.

While subjective, the quality of evidence assessed with the GRADE framework indicated that the majority of studies judged as of high quality were focused on CXR, with only one study focused on CT. This again confirms the limited good quality evidence on symptomatic patients in primary care.

We did not conduct a meta-analysis. This was not possible due to the heterogeneity of the included studies; even for studies reporting on the same imaging modality, there were great differences in the diagnostic pathways used, definition of positive cases and populations selected. As mentioned, some of the studies also had overlapping populations.

### Relevance to practice

Intrathoracic cancer diagnosis is challenging and not straightforward, with guidelines offering variable recommendations. This review summarised the evidence on imaging performance for intrathoracic cancers in primary care and highlighted the need for further research in primary care, including future studies with reliable sample sizes and well-defined populations.

For symptomatic patients presenting in primary care, CXR is the most common first imaging investigation. In lesions affecting the thoracic wall (including pleurae), US has also been used, although CT can provide not only information on the areas that cannot be visualised by US, but also better information overall on the presence and nature of intrathoracic lesions than US.

Specifically, the NICE guidelines on lung cancer^7^ offer limited imaging options to primary care practitioners. The guidelines on recognition and referral for lung cancer (NG12) only include recommendations for CXR based on specific symptoms, as well as urgent referral for unexplained haemoptysis or an abnormal CXR. More advanced imaging, such as CT, MRI, PET-CT and scintigraphy, is usually used in secondary care, and rarely, the first imaging technique to be used when intrathoracic cancer is initially suspected in primary care.

### Conclusion

There was limited information on the diagnostic performance of usual imaging techniques when used in unselected primary care settings for the diagnosis of intrathoracic cancer in symptomatic patients.

More studies need to focus on the evaluation of such techniques (CT in particular) in the general population presenting in primary care, where the prevalence is relatively low. A better understanding of their performance could inform more efficient detection strategies for intrathoracic cancers in primary care, leading to earlier detection and, ultimately, better outcomes.

## Supplementary material

10.1136/bmjopen-2024-091435 online supplemental file 1

## Data Availability

All data relevant to the study are included in the article or uploaded as supplementary information.
