# Supplementary material for: Evaluation of imaging techniques for early detection of intrathoracic cancers in symptomatic patients in primary care: a systematic review
Source: BMJ Open. 2025 Aug 16;15(8):e091435. doi: 10.1136/bmjopen-2024-091435 (PMC12359469; doi:10.1136/bmjopen-2024-091435)
Supplement: online supplemental file 1 [file bmjopen-15-8-s001.docx]

**Appendix 1: Search strategy**

**Ovid MEDLINE(R) and Epub Ahead of Print, In-Process, In-Data-Review & Other Non-Indexed Citations, Daily and Versions**1946 to December 02, 2022 (searches carried out on 17/01/2023)

| 1 | exp respiratory tract neoplasms/ or exp thymus neoplasms/ or exp thymoma/ or exp pleural neoplasms/ or exp tracheal neoplasms/ | 339268 |
| --- | --- | --- |
| 2 | exp lung neoplasms/ or exp bronchial neoplasms/ or exp carcinoma, bronchogenic/ or exp adenocarcinoma of lung/ or exp mesothelioma, malignant/ or exp multiple pulmonary nodules/ or exp pancoast syndrome/ or exp carcinoma, non small-cell lung/ or exp small cell lung carcinoma/ or exp carcinoma, large cell/ | 268832 |
| 3 | ((lung or "non small cell" or "nonsmall cell" or NSCLC or "large cell" or pulmonary or thoracic or trachea* or thym* or mediastin* or bronch*) adj2 (canc* or carcino* or tumo?r* or neoplasm* or sarcom* or nodul* or "squamous cell")).ti,ab. | 271795 |
| 4 | (mesothelioma or thymoma or "central carcinoid*" or "peripheral carcinoid*").ti,ab. | 25870 |
| 5 | 1 or 2 or 3 or 4 | 441816 |
| 6 | exp Diagnostic Imaging/ or (((CT or CAT) adj3 scan$) or ((computer$ adj3 tomogra$) and scan$) or (tomogra$ or helix or helical or spiral$ or spiro$) or (chest x-ray) or (MRI) or (Digital Chest Tomosynthesis) or ultrasound).ti,ab,ot,kw. | 3468678 |
| 7 | (Sensitivit* or Specificit* or ROC Curve* or receiver operat* characteristic or ROC or predictive value* or PPV or NPV or false negative* or false positive* or true negative* or true positive* or accurac* or area under the curve* or AUC* or AUROC* or performance* or discriminat* ability or discriminat* or (diagnos* adj2 (mistake* or error*))).ti,ab. | 3127560 |
| 8 | exp "Sensitivity and Specificity"/ | 643135 |
| 9 | 7 or 8 | 3424817 |
| 10 | 5 and 6 and 9 | 16657 |
| 11 | exp animals/ not (exp animals/ and humans/) | 5082768 |
| 12 | 10 NOT 11 | 16409 |
| 15 | limit 14 to yr="2000 -Current" | **14259** |

Embase

**Embase**1996 to 2023 January 13 (searches carried out on 17/01/2023)

| 1 | exp *lung tumor/ or exp *respiratory tract cancer/ or exp *lung sarcoma/ or exp *Pancoast tumor/ or exp *lung carcinoma/ | 282920 |
| --- | --- | --- |
| 2 | exp *thymoma/ or exp *thymus carcinoma/ or exp *thymic neoplasm/ or exp *pleura mesothelioma/ or exp *trachea tumor/ or exp *trachea carcinoma/ or exp *trachea cancer/ | 19662 |
| 3 | ((lung or "non small cell" or "nonsmall cell" or NSCLC or "large cell" or pulmonary or thoracic or trachea* or thym* or mediastin* or bronch*) adj2 (canc* or carcino* or tumo?r* or neoplasm* or sarcom* or nodul* or "squamous cell")).ti,ab. | 394980 |
| 4 | (mesothelioma or thymoma or "central carcinoid*" or "peripheral carcinoid*").ti,ab. | 35459 |
| 5 | 1 or 2 or 3 or 4 | 496307 |
| 6 | exp Diagnostic Imaging/ or (((CT or CAT) adj3 scan$) or ((computer$ adj3 tomogra$) and scan$) or (tomogra$ or helix or helical or spiral$ or spiro$) or (chest x-ray) or (MRI) or (Digital Chest Tomosynthesis) or ultrasound).ti,ab,ot,kw. | 2043384 |
| 7 | exp *diagnosis/ | 1971223 |
| 8 | 6 or 7 | 3361446 |
| 9 | (Sensitivit* or Specificit* or ROC Curve* or receiver operat* characteristic or ROC or predictive value* or PPV or NPV or false negative* or false positive* or true negative* or true positive* or accurac* or area under the curve* or AUC* or AUROC* or performance* or discriminat* ability or discriminat* or (diagnos* adj2 (mistake* or error*))).ti,ab. | 3854537 |
| 10 | exp *"sensitivity and specificity"/ | 2724 |
| 11 | exp *diagnostic error/ or exp *false negative result/ or exp *false positive result/ or exp *missed diagnosis/ | 13995 |
| 12 | exp *area under the curve/ | 10087 |
| 13 | 9 or 10 or 11 or 12 | 3863905 |
| 14 | 5 and 8 and 13 | 22824 |
| 15 | limit 14 to yr="2000 -Current" | **21053** |

**Web of Science**

| 1 | TI=(((lung or "non small cell" or "nonsmall cell" or NSCLC or "large cell" or pulmonary or thoracic or trachea* or thym* or mediastin* or bronch*) NEAR/2 (canc* or carcino* or tumo?r* or neoplasm* or sarcom* or nodul* or "squamous cell"))) | 210,051 |
| --- | --- | --- |
| 2 | AB=(((lung or "non small cell" or "nonsmall cell" or NSCLC or "large cell" or pulmonary or thoracic or trachea* or thym* or mediastin* or bronch*) NEAR/2 (canc* or carcino* or tumo?r* or neoplasm* or sarcom* or nodul* or "squamous cell")) ) | 198,826 |
| 3 | TI=((mesothelioma or thymoma or "central carcinoid*" or "peripheral carcinoid*") ) | 19,516 |
| 4 | AB=((mesothelioma or thymoma or "central carcinoid*" or "peripheral carcinoid*") ) | 16,070 |
| 5 | #1 OR #2 OR #3 OR #4 | 322,609 |
| 6 | TI=( (Diagnostic Imag*) OR ((CT OR CAT) adj3 scan$) OR ((computer$ adj3 tomogra$) AND scan$) OR (tomogra$ OR helix OR helical OR spiral$ OR spiro$) OR (chest x-ray) OR MRI OR (Digital Chest Tomosynthesis) OR (ultrasound)) | 386,907 |
| 7 | AB=( (Diagnostic Imag*) OR ((CT OR CAT) adj3 scan$) OR ((computer$ adj3 tomogra$) AND scan$) OR (tomogra$ OR helix OR helical OR spiral$ OR spiro$) OR (chest x-ray) OR MRI OR (Digital Chest Tomosynthesis) OR (ultrasound)) | 908,824 |
| 8 | #7 OR #6 | 1,070,679 |
| 9 | TI=(diagnos* or detect* or "early diagnos*") | 1,804,978 |
| 10 | AB=(diagnos* or detect* or "early diagnos*") | 5,867,260 |
| 11 | #9 OR #10 | 6,625,982 |
| 12 | #8 AND #11 | 398,349 |
| 13 | TI=(Sensitivit* or Specificit* or "ROC Curve*" or "receiver operat* characteristic" or ROC or "predictive value*" or PPV or NPV or "false negative*" or "false positive*" or "true negative*" or "true positive*" or accurac* or "area under the curve*" or AUC* or AUROC* or performance* or "discriminat* ability" or discriminat* or (diagnos* NEAR/2 (mistake* or error*))) | 1,584,438 |
| 14 | (Sensitivit* or Specificit* or "ROC Curve*" or "receiver operat* characteristic" or ROC or "predictive value*" or PPV or NPV or "false negative*" or "false positive*" or "true negative*" or "true positive*" or accurac* or "area under the curve*" or AUC* or AUROC* or performance* or "discriminat* ability" or discriminat* or (diagnos* NEAR/2 (mistake* or error*)))  (Abstract) | 7,294,868 |
| 15 | #14 OR #13 | 7,937,301 |
| 16 | #15 AND #12 AND #5 | 3,154 |
| 17 | #16                         DOP=(2000-01-01/2023-01-17) | 52,706,204 |
| 18 | #17 AND #16 | 2,965 |

**Appendix 2. Data extraction**

| **Study** | **Country of population of interest** | **Study aims** | **Study design** | **Sample source** | **Inclusion criteria** | **Numbers included** | **Age** | **Sex** | **Exclusions** | **High-risk population?** | **Type of cancer investigated** |
| --- | --- | --- | --- | --- | --- | --- | --- | --- | --- | --- | --- |
| Bai 2022 | China | to study the diagnostic effect of high-resolution reorganization of medical images based on deep CNN in lung cancer | prospective cohort | prospective selection of 74 patients with highly suspected lung cancer who were in the hospital from January 2017 to January 2021 | high suspicion of lung cancer: all patients clinically accompanied by cough, vocal tearing, expectoration, and blood in sputum | 74 | 52 ±6 years | 44 males and 30 females | NR | N | LC |
| Bhartia 2021 | UK | to establish the accuracy of the CXR in the investigation of all primary intrathoracic malignancies amongst symptomatic adults over the age of 50-yrs. Secondary aims were to evaluate how a history of smoking alters the test characteristics, the role of CXR follow-up, and whether the reported presence of abnormalities for which no follow-up is advised predict the presence of malignancy | prospective cohort | a cohort of consecutive “self-request” CXR studies prospectively collated between January 2011 and October 2016 as part of the National Awareness and Early Diagnosis Initiative (NAEDI) | symptoms persisting for at least 3 weeks (cough, haemoptysis, shortness of breath, chest pain, change in voice or loss of weight) and age over 50-yrs | 8948 | Over 50 years | 4415 male and 4533 female | CXR in the previous 3 months | N | all intrathoracic cancers |
| Bradley 2021 | UK | to determine the sensitivity and specificity of CXR for lung cancer in patients with symptoms aged >50 years, who requested the investigation; • to estimate the risk (PPVs) of being diagnosed with lung cancer within 1 year and 2 years following a negative CXR result for a range of symptoms and symptom combinations; and • to determine whether the symptoms associated with lung cancer are different in those who had a positive CXR result compared with those who had a negative CXR result | prospective cohort | routinely collected data that had been obtained between January 2011 and October 2016 from an SR-CXR service at Leeds Teaching Hospitals NHS Trust | patients with relevant symptoms: cough, haemoptysis, dyspnoea, chest pain, weight loss, and change in voice | 8996 | Over 50 years | 4441 male and 4555 female | history of lung cancer | N | LC |
| Clin 2009 | France | to compare, in a cohort of asbestos-exposed workers, the sensitivity and the specificity of low-radiation helical chest CT scan with chest radiograph for the biennial screening of bronchopulmonary cancer, according to the size of detected nodules | prospective cohort | asymptomatic patients 50-75 years old with "important" asbestos exposure, examined between 1st January 2000 and 31st December 2006 | age 50-75 at first examination; "important" asbestos exposure; asymptomatic for cancer at the time of inclusion | 972 (1230 screening procedures) | 61.29 (6.56) male; 61.45 (6.97) female | 920 males, 52 females | patients without 2-year follow up | Y, asbestos exposure | LC |
| Felten 2014 | Germany | to test whether that nonautomated sputum cytology is be particularly useful for the diagnosis of centrally located tumours, whereas LDSCT is more capable of detecting tumours in the peripheral parts of the lung | prospective cohort | patients with asbestos exposure | high risk patients within those with exposure to asbestos, <75 years | 187 | 65.8 ±5.8 years | NR | no sputum results, refused to partake in either screen or results not usable | Y, asbestos  exposure | LC |
| Heye 2012 | Germany | to assess the accuracy of lesion detection by MRI compared to CT that can be realistically expected in the clinical routine | prospective cohort | patients with suspected thoracic malignancy and clinical indication of diagnostic CT imaging of the chest | suspected thoracic malignancy and clinical indication of diagnostic CT imaging of the chest | 28 | median 66 | 16 male, 12 female | contraindications to CT or MRI | N | thoracic - primary malignancies and lung metastases |
| Maiter 2023 | UK | to evaluate the performance of Auto Lung Nodule Detection system at detecting lung nodules on chest radiographs seen in routine practice in a tertiary UK centre | retrospective | CXR acquired between July 2020 and 26 February 2021 at the Sheffield Teaching Hospitals, UK | CXR: 1) on adult patients, 2) requested by the GP, 3) acquired in the posterior-anterior projection, 4) on one of three GC85A digital radiography systems running the software tool | 5722 CXR from 5592 patients (only 2.3% had more than one CXR) | 59 years (IQR 46–72 years) | 53.8% female | none | N | LC |
| Rinaldi 2020 | Italy | to assess the role of lung ultrasound in the clinical diagnostic algorithm of respiratory diseases | prospective cohort | consecutive patients admitted for acute respiratory symptoms | acute respiratory symptoms (cough, chest tightness and thoracic pain) | 509 | median 71 | 52.5% males | known history of respiratory illness, patients admitted for thoracic trauma and those younger than 18 years | N | LC |
| Traill 2001 | UK | to determine the sensitivity and specificity of contrast-enhanced CT for the detection of pleural malignancy in patients referred for the diagnosis of a suspected malignant pleural effusion, using histopathology, cytology and clinical follow-up as gold standards | prospective cohort | consecutive patients with suspected malignant pleural effusion | suspected malignant pleural effusions | 40 | median 69 (45-89) | 28 male, 12 female | NR | N | Malignant pleural effusions |
| Tsim 2017 | UK | to assess relative to ‘real-life’ factors including use of early arterial-phase contrast enhancement (by CT pulmonary angiography (CTPA)) and non-specialist radiology reporting | retrospective | consecutive patients recruited prospectively with suspected pleural malignancy | suspected pleural malignancy (defined by a unilateral pleural effusion or pleural mass lesion); sufficient fitness for at least a pleural aspiration and informed written consent | 315 | median 74 years (65-79) | 204 males | inter-costal chest drain in-situ, or one within the preceding 3 months; incomplete (<6 months) follow-up | Y (only some of the patients) asbestos exposure | Pleural malignancy, including mesothelioma and secondary malignancies |
| Wang 2007 | China | to evaluate the clinical value of tomographic 99mTc-octreotide acetate (hereafter, 99mTc-octreotide) scintigraphy in the detection of patients with suspected lung cancer in comparison with that of 18F-FDG dual-head coincidence imaging (DHC) | prospective cohort | consecutive patients with suspected lung cancer | suspected lung cancer (no further details) | 44 | 62 ±10 years | 31 male, 13 female | NR | N | LC |
| Yang 2022 | China | to investigate the diagnostic capabilities of high-frequency B-mode ultrasound (US) and contrast-enhanced US (CEUS) in terms of differentiating between benign and malignant pleural diseases | prospective cohort | consecutive patients referred for pleural effusion or pleural thickening | pleural thickening (≥3 mm) or a pleural lesion, a diagnosis of pleural pathology, no prior anti-tumor treatment, patient consent, and age >18 years | 50 | median 58 years | 37 male, 13 female | any history of hypersensitivity to a CEUS agent, an inability to undergo CEUS because of tachypnea, a lesion located in the lung or chest wall, unclear US images, or insufficient data | N | Pleural malignancy |
| Zhou 2020 | China | to evaluate the effect of GdCho‑lenvatinib nanoparticles contrast‑PET/CT (GdCho‑Len‑PET) in the diagnosis and treatment planning of a cohort of patients suspected of having lung cancer | prospective cohort | patients with suspected lung cancer from the Dongzhimen Hospital of Beijing University of Traditional Chinese Medicine (Beijing, China) between May 2016 and September 2017 | i) age ≥25 years; and ii) individuals who were able to provide informed consent for participation | 172 | 47.6 years (36-60) | 86 male, 86 female | i) Patients with cancer history; ii) patients with pulmonary infarction; iii) patients who had been diagnosed with acute respiratory disease within 6 months; iv) pregnant or lactating females; and v) patients with infection suspected to cause coughs | N | LC |

| **Study** | **Index test** | **Prior imaging** | **Setting** | **Definition of positives** | **Comparator** | **Ref std** | **Ref std setting** | **Timing of ref std** | **Area under curve** | **Conclusions** |
| --- | --- | --- | --- | --- | --- | --- | --- | --- | --- | --- |
| Bai 2022 | convolutional neural network analysis of multi-slice spiral CT and MRI | NR, assume none | hospital | Presence of lung cancer | MRI, CT | biopsy | hospital | NR | NR | Both CT and MRI images enhanced by deep CNN have high accuracy |
| Bhartia 2021 | CXR | N | hospital | 1. abnormal report directly initiating CT; 2.abnormal report with recommendation of further plain film evaluation; subsequent study initiates CT; CT request references plain film abnormality; 3. abnormal report with recommendation of further clinical evaluation; subsequent study initiates CT; CT request references plain film abnormality | N | malignancies recorded in cancer registries within a one- or two-year post-test period | hospital | up to 2-year (registry data) from CXR | NR | A positive CXR is highly suggestive of malignancy, but a negative CXR "does not conclusively exclude" malignancy |
| Bradley 2021 | CXR | N | hospital | 1. suspicion of lung cancer identified/urgent investigation indicated; 2. abnormality identified/non urgent investigation indicated | combination of CXR and symptoms | database of all patients who received a multidisciplinary team-approved diagnosis of lung cancer in LTHT between 2011 and 2018 | hospital | between 1 and 2 years after CXR | NR | A negative CXR suggests v low chance of LC with most symptoms, except haemoptysis |
| Clin 2009 | CXR, CT | Y, some (more than one screen) | NR, presumably secondary care | localised pulmonary opacity, non-linear, unique or multiple, but with no more than six nodules, with an average diameter of over 2 mm in tomodensitometry (TDM), devoid of any radiological marker of benignity (such as a totally calcified aspect, or, on the CT scan, clearly confirmed partly fatty density and/or established stability over time) | CXR | histology (for +ve) or 2-year follow up (for -ve) | hospital | Up to 2 yrs after the screen | NR | CT had better sensitivity compared to CXR in detecting lung cancer |
| Felten 2014 | LDCT | Y, CXR/LDCT | NR, presumably secondary care | "Suspicious lesion" | Cytology, LDCT+cytology | occupational registry and death certificates | hospital | NR | NR | - |
| Heye 2012 | MRI | unclear | NR, presumably secondary care | lung lesion detected | CT | contrast CT, surgery and biopsy if performed | hospital | same day | NR | MRI of the chest is as reliable as CT in detection and size depending clinical T-staging of primary thoracic malignancies |
| Maiter 2023 | CXR Auto Lung Nodule Detection (ALND) software | unclear, although 2.3% of patients had more than one CXR | hospital | binary assessment for the presence of a suspicious lung nodule (i.e., “yes” or “no”) as assessed by the software |  | 1) radiologists’ reports  2) records of the MTD up to 6 months from CXR acquisition | hospital | up to 6 months from index test | NR | The software demonstrated low PPV values and underperformed compared with radiologists’ reports. This severely limits the potential utility of the software and makes it unsuitable for a primary care population. |
| Rinaldi 2020 | LUS | NR | hospital | signs of lesions | CXR, CT | discharge dx | hospital | NR | NR (only reported AUROC including non-cancer diagnoses) | LUS findings were highly concordant with the cancer diagnostic (41/44 cases), however this was mostly due to the frequent finding of indirect signs such as pleural effusion or thickening, large areas of atelectasis and secondary peripheral lesions |
| Traill 2001 | CE-CT | NR | hospital | nodular or irregular pleural thickening or smooth thickening greater than 1 cm | N | histology or follow up | hospital | >1 yr after CT or to death | NR | CT criteria for differentiating malignant/benign pleural disease |
| Tsim 2017 | CE-CT | NR | hospital | abnormal images, suggestive of malignancy | N | cytology/histology or follow up | hospital | follow-up > 6 months | NR | CT in routine practice is not good enough to confirm/exclude pleural malignancy |
| Wang 2007 | scintigraphy (99mTc-Octreotide SPECT) | Y, CXR, CT | hospital | areas of increased tracer uptake were considered positive for malignancy | 18F-FDG SPECT | histology or follow up | hospital | NR | NR | Scintigraphy has comparable accuracy to PET-CT |
| Yang 2022 | contrast-enhanced ultrasound (CEUS) | NR | hospital | NR |  | biopsy for all malignant; biopsy or follow-up for benign | hospital | NR | for pleural thickening: B-mode US AUC 0.819 (95% CI: 0.696 to 0.942);  high-frequency CEUS AUC of 0.848 (95% CI: 0.740 to 0.957) | both B-mode US and CEUS were able to clearly distinguish between benign and malignant thickened pleurae |
| Zhou 2020 | GdCho‑Len‑PET | NR | hospital | NR | GdCho‑PET | histopathology | hospital | NR | NR | GdCho‑Len‑PET presented with higher accuracy and sensitivity compared with GdCho‑PET in diagnosing patients with lung cancer |

Note: NR, not reported

Appendix 3. Accuracy data extracted from the included papers

| **Study** | **Outcome** | **Prevalence** | **Imaging test** | **PPV**  **% (95% CI)** | **NPV**  **% (95% CI)** |
| --- | --- | --- | --- | --- | --- |
| CXR | | | | | |
| Bhartia 2021 | All intrathoracic malignancies | 1.5% | CXR 1y f-u | 20.4  (16.8–23.9) | 99.6  (99.5–99.8) |
| Bhartia 2021 | All intrathoracic malignancies | 1.5% | CXR 2y f-u | 20.4  (16.8–23.9) | 99.2  (99.0–99.5) |
| Bradley 2021 | Lung cancer | 1.3% | CXR 1y f-u | 9.0  (7.2 - 10.8) | 99.7  (99.5 - 99.8) |
| Bradley 2021 | Lung cancer | 1.7% | CXR 2y f-u | 10.2  (8.3 - 12.3) | 99.3  (99.1 - 99.5) |
| Maiter 2023 | Lung cancer | 1.6% | CXR ALND AI against MTD | 5.6  (4.8 –6.6) | 99.2  (99.0 –99.4) |
| Clin 2009 | Lung nodules >2mm | 2.0% | CXR^1^ | 12.5  (5.6 – 23.2) | 98.6  (97.8 – 99.2) |
| CT |  |  |  |  |  |
| Bai 2022 | Lung cancer | 62.2% | AI enhanced CT | 95.7  (85.2 – 99.5) | 92.9  (76.5 – 99.1) |
| Clin 2009 | Lung nodules >2mm | 2.0% | CT^1^ | 7.0  (4.3 – 10.6) | 99.6  (98.9- 99.9) |
| Felten 2014 | Lung cancer | 6.4% | LDSCT | 64.7  (38.3 – 85.8) | 99.4  (96.8 – 100.0) |
| Felten 2014 | Lung cancer | 6.4% | LDSCT + sputum cytology | 60.0  (36.1 – 80.9) | 100  (97.8 – 100.0) |
| Traill 2001 | Malignant pleural effusions | 80% | Contrast enhanced CT | 84.4  (67.2 – 94.7) | 37.5  (8.5 – 75.5) |
| Tsim 2017 | Pleural malignancy, including mesothelioma and secondary malignancies | 61.9% | Contrast enhanced CT | 83.2  (75.9 – 89.0) | 54.5  (46.9 – 62.0) |
| MRI |  |  |  |  |  |
| Bai 2022 | Lung cancer | 62.2% | AI enhanced MRI | 91.3  (79.2 – 97.6) | 85.7  (67.3 – 96.0) |
| PET-CT |  |  |  |  |  |
| Wang 2007 | Lung cancer | 70.5% | ^18^FDG PET-CT | 81.6  (65.7 – 92.3) | 100  (54.1 – 100) |
| Zhou 2020 | Lung cancer | 79.1% | GdCho‑Len-PET | 86.2  (79.7 – 91.2) | 75.0  (50.9 – 91.3) |
| Zhou 2020 | Lung cancer | 79.1% | GdCho-PET | 78.5  (65.9 – 91.4) | 19.0  (65.9 – 91.4) |
| Ultrasound |  |  |  |  |  |
| Rinaldi 2020 | Lung cancer | 8.6% | Ultrasound | 100.0  (91.4 – 100.0) | 99.4  (98.1 – 99.9) |
| Scintigraphy |  |  |  |  |  |
| Wang 2007 | Lung cancer | 70.5% | Scintigraphy (^99m^Tc-Octreotide) | 88.6  (73.3 – 96.8) | 100  (96.8 – 100) |

Note: 1-y f-u – one year follow-up; 2-y f-u – two-year follow-up; ^1^ – lesions >2mm; MTD = multidisciplinary team

Appendix 4. Risk of bias (QUADAS-2)

| Study | Was a consecutive or random sample of patients enrolled? | Was a case-control design avoided? | Did the study avoid inappropriate exclusions? | Could the selection of patients have introduced bias? | Is there concern that the included patients do not match the review question? | Were the index test results interpreted without knowledge of the results of the reference standard? | If a threshold was used, was it pre-specified? | Could the conduct or interpretation of the index test have introduced bias? | Is there concern that the index test, its conduct, or interpretation differ from the review question? | Is the reference standard likely to correctly classify the target condition? |
| --- | --- | --- | --- | --- | --- | --- | --- | --- | --- | --- |
| Bai 2022 | NR | Y | Unclear | Unclear | Unclear | Unclear | Y | Unclear | Unclear | Y |
| Bhartia 2021 | Y | Y | Y | Low | Low | Y | Y | Low | Low | Y |
| Bradley 2021 | Y | Y | Y | Low | Low | Y | Y | Low | Low | Unclear |
| Clin 2009 | Unclear | Y | Y | Unclear | Unclear | Y | Y | Low | Low | Unclear |
| Felten 2014 | N | Y | Y | Unclear | Low | Y | Y | Low | Low | Y |
| Heye 2012 | Unclear | Y | Unclear | Unclear | High | Y | Y | Low | Low | Unclear |
| Maiter 2023 | Unclear | Y | Y | Low | Low | Y | Y | Low | Low | Y |
| Rinaldi 2020 | Y | Y | Y | Low | High | Y | Y | Unclear | High | Y |
| Traill 2001 | Y | Y | Unclear | Unclear | Low | Y | Y | Low | Low | Y |
| Tsim 2017 | Y | Y | Y | Low | Low | Y | Y | Low | Low | Y |
| Wang 2007 | Y | Y | Unclear | Unclear | Unclear | Y | Y | Low | Low | Unclear |
| Yang 2022 | Y | Y | Y | Low | Low | Y | Unclear | Unclear | High | Y |
| Zhou 2020 | Unclear | Y | Y | Unclear | Unclear | Y | N | High | High | Y |

| Study | Were the reference standard results interpreted without knowledge of the results of the index test? | Could the reference standard, its conduct, or its interpretation have introduced bias? | Is there concern that the target condition as defined by the reference standard does not match the review question? | Was there an appropriate interval between index test(s) and reference standard? | Did all patients receive a reference standard? | If not all pts received ref std, how identified those who did receive ref std? | Did patients receive the same reference standard? | Were all patients included in the analysis? | Could the patient flow have introduced bias? | Was the study method pre-specified? |
| --- | --- | --- | --- | --- | --- | --- | --- | --- | --- | --- |
| Bai 2022 | Unclear | Unclear | Unclear | Unclear | Y | NA | Y | Y | Low | N |
| Bhartia 2021 | Y | Low | Low | Unclear | Y | NA | Y | Y | Unclear | Y |
| Bradley 2021 | Unclear | Unclear | Unclear | Unclear | Unclear | NA | Y | Y | Unclear | Y |
| Clin 2009 | Unclear | Unclear | Unclear | Y | Y | NA | N | Y | Unclear | Y |
| Felten 2014 | Unclear | High | Low | Unclear | Y | NA | N | Unclear | Unclear | Y |
| Heye 2012 | Unclear | Unclear | High | Y | Y | NA | Unclear | Y | Low | Y |
| Maiter 2023 | Y | Low | Low | Y | Y | NA | Y | Y | Low | Y |
| Rinaldi 2020 | Unclear | Unclear | Low | Y | Y | NA | Unclear | Y | Unclear | Y |
| Traill 2001 | Unclear | Low | Low | Y | Y | NA | N | Y | Low | N |
| Tsim 2017 | Unclear | Unclear | High | Y | Y | NA | N | Y | Low | Y |
| Wang 2007 | Unclear | Unclear | Unclear | Unclear | Y | NA | N | Y | Unclear | Y |
| Yang 2022 | Unclear | Low | High | Y | Y | NA | N | Y | Low | Y |
| Zhou 2020 | Unclear | Low | Low | Y | Y | NA | N | Y | Low | Y |

Appendix 5. GRADE assessments

| **Paper** | **RoB** | **Consistency** | **Directness** | **Precision** | **Publication bias** | **Quality of evidence** |
| --- | --- | --- | --- | --- | --- | --- |
| CXR | | | | | | |
| Bhartia 2021 | Low risk | Consistent | Direct | Precise | Unknown | ⊕⊕⊕⊕⊝ High |
| Bradley 2021 | Low risk | Consistent | Direct | Precise | Unknown | ⊕⊕⊕⊕⊝ High |
| Maiter 2023 | Low risk | Consistent | Direct | Precise | Unknown | ⊕⊕⊕⊕⊝ High |
| Clin 2009 | Unknown risk | Unknown | Indirect | Imprecise | Unknown | ⊝⊝⊝⊝⊝ Low |
| CT | | | | | | |
| Bai 2022 | Unknown risk | Consistent | Direct | Precise | Unknown | ⊝⊕⊕⊕⊝ Low |
| Felten 2014 | Medium risk | Consistent | Direct | Precise | Unknown | ⊝⊕⊕⊕⊝ Moderate |
| Traill 2001 | Low risk | Unknown | Direct | Imprecise | Unknown | ⊕⊝⊕⊝⊝ Moderate |
| Tsim 2017 | Low risk | Consistent | Direct | Precise | Unknown | ⊕⊕⊕⊕⊝ High |
| MRI | | | | | | |
| Heye 2012 | Low risk | Unknown | Indirect | Imprecise | Unknown | ⊕⊝⊝⊝⊝ Low |
| Bai 2022 | Unknown risk | Unknown | Indirect | Imprecise | Unknown | ⊝⊝⊝⊝⊝ Low |
| PET-CT | | | | | | |
| Yang 2022 | Low risk | Consistent | Direct | Precise | Unknown | ⊕⊕⊕⊕⊝ High |
| Zhou 2020 | Medium risk | Consistent | Direct | Precise | Unknown | ⊝⊕⊕⊕⊝ Moderate |
| Ultrasound | | | | | | |
| Rinaldi 2020 | Unknown risk | N/A | Direct | Precise | Unknown | ⊝ ⊕⊕⊝ Moderate |
| Scintigraphy | | | | | | |
| Wang 2007 | Unknown risk | N/A | Direct | Imprecise | Unknown | ⊝ ⊕⊝⊝ Low |
