# Supplementary material for: Evaluation of imaging techniques for early detection of intrathoracic cancers in symptomatic patients in primary care: a systematic review
Source: BMJ Open. 2025 Aug 16;15(8):e091435. doi: 10.1136/bmjopen-2024-091435 (PMC12359469; doi:10.1136/bmjopen-2024-091435)
Supplement: Supplementary file 2 [file bmjopen-2024-091435.reviewer_comments.pdf]

PEER REVIEW HISTORY

BMJ Open publishes all reviews undertaken for accepted manuscripts. Reviewers are asked to complete a checklist review form and are provided with free text boxes to elaborate on their assessment. These free text comments are reproduced below.

ARTICLE DETAILS

Title (Provisional)

Evaluation of imaging techniques for early detection of intrathoracic cancers in symptomatic patients in primary care: a systematic review

Authors

Grigore, Bogdan; Peters, Jaime L; Hamad, Wasim; Calanzani, Natalia; Asare, Lauren; Walter, Fiona M; Neal, Richard

---

VERSION 1 - REVIEW

**Reviewer**	**1**
**Name**	**Lim, Gilbert**
**Affiliation**	**National University of Singapore, School of Computing**
**Date**	**13-Sep-2024**
**COI**	**N/A**

This manuscript is a systematic review evaluating imaging techniques for the early detection of intrathoracic cancers in symptomatic patients in primary care. From over 30,000 candidate papers retrieved from various databases, a relatively small number (13 studies) were included. This resulted in the conclusion that more such studies in primary care settings (with low prevalence) were needed.

Some issues might be addressed:

1. In the Methods section, the methodology for quality assessment (QUADAS-2) might be described in further detail. In particular, was arbitration with multiple reviewers also done as for screening and data extraction? The qualifications of the various reviewers (e.g. medical training, years of experience) might also be stated if possible, for all steps in the process.

2. In the Methods section, it is claimed that "A number of meetings were organised with patient representatives throughout the project. Patient representatives were invited to provide feedback and suggestions on both the study protocol and the study results, as they became available". Details about these meetings (including any processes such as Delphi consensus), and updates to the protocol/study resulting from such meetings might be stated.

3. Figure 1 might be rendered as a high-definition image on a single page, instead of being divided across page boundaries as currently.

4. For trial outcomes in Table 3, aggregate statistics such as AUROC/AUPRC might be included, if possible.

5. Vertical alignment in Appendix 2 might be standardized.

6. NR in Appendix 3 might be clarified.

7. Some minor spelling/grammatical errors (e.g. "by each reviewe" in the Data Extraction subsection, "An update search" in the Results subsection) might be corrected.

Reviewer 2

Name Kim, Grace

Affiliation University of California Los Angeles Jonathan and Karin Fielding School of Public Health, Biostatistics

Date 30-Sep-2024

COI None

This is an important study using a systematic search of the literature to identify diagnostic performance of imaging in primary care and studies reporting on impact of diagnostic imaging in primary care, as well as studies reporting the use of artificial intelligence.

This study includes papers which are relevant for primary care, where participants were suspected intrathoracic cancer and reported on at least one diagnostic performance. The potential bias and the limited of variabilities may be difficulty to rely on the finding of the results for the study question.

In the flow chart from Figure 1, 130 papers were assessed in full-text screening. Thirteen papers were included in the final review.

Here are major comments

(1) Lack of clarification from 130 papers in full-text screening to 13 papers in terms of the detailed methods used to assess risk of bias in the included studies, including details of the tool(s) used, whether they worked independently and the method of training before start review, and, if applicable, details of automation tools used in the process

-what are the major and minor excluded criteria of 117 papers? Although Figure 1 explains the reasons, it was a surprise that there were no imaging studies, and no cancer studies.

(2) Recommend add forest plot from the meta-study analysis for sensitivity, specificity, PPV, NPV, and possibly accuracy.

(3) it is uncertain whether two readers are enough to review addressing evidence on the performance of imaging techniques that could aid the detection of intrathoracic cancers in low prevalence settings. When two indepdent readers disagree (if it happened), is there adjudication or consensus process?

Minor

(1) Inconsistent format of references

(2) In the last paragraph of Discussion, "We identified only one report evaluating the use of an AI system in a primary care context…. This outcome highlights the broader issue of many AI imaging tools, where datasets used for training and validation do not reflect the prevalence of the general population."

AI tools can be presented in the multiple papers over time. Do you think that your searching method can cover the results over time? It is quite a surprise that this search found only one paper out of 24,756 screening.

**Reviewer**	**3**
**Name**	**Ambrosini, Robert**
**Affiliation**	**University of Rochester Medical Center**
**Date**	**18-Dec-2024**
**COI**	**None**

- This paper focuses on the utilization of imaging to detect intrathoracic cancers in primary care settings. The authors conducted a systematic search of the literature to identify studies that looked at imaging including x-ray, CT, MRI, ultrasound, PET, and scintigraphy for detection of intrathoracic malignancy in low prevalence settings.

- The paper itself is well-organized and well-written. The Introduction is concise but informative briefly covering each of the included imaging modalities. The Methods section describes the authors approach to the literature search and data processing in satisfactory detail and the Results section includes relatively thorough summaries of each of the relevant papers that were found by the authors during this literature search.

- The results of the paper, as stated by the authors, are that only a limited number of relevant studies were available and that there is a need for more studies looking at the value of imaging for detection of intrathoracic malignancy in the primary care setting

- As stated by the authors, they are unable to provide any significant synthesis of the results of their literature search due to an overall paucity of relevant papers that were discovered. The Discussion section instead focuses upon the challenges of intrathoracic malignancy detection in the primary care setting, which seems appropriate

- The provided Figures/Tables include breakdowns of the authors' search process, which are a positive addition to the manuscript. No medical imaging is provided with this manuscript, however this seems acceptable given the literature search nature of this paper.

**Reviewer**	**4**
**Name**	**Henderson, Jillian**
**Affiliation**	**Kaiser Permanent Center for Health Research, Northwest**
**Date**	**15-Apr-2025**
**COI**	**No competing interests.**

The authors present a well-written systematic review of the evidence on different imaging tests used to detect intrathoracic cancers in primary care settings. There are a few areas where the review could be strengthened.

1. The study would benefit from the addition of a summary of evidence table that includes certainty ratings for the test performance results with the approach to evaluating certainty outlined in the methods section (E.g., GRADE, EHC https://effectivehealthcare.ahrq.gov/products/methods-guidance-tests-grading/methods).

Although the authors describe limitations in the evidence throughout the manuscript, a valuable contribution of systematic reviews is the application of a clear procedure for making determinations about overall certainty.

2. The review focuses on studies of the test performance of imaging modalities among symptomatic patients presenting in primary care. Presumably this is the focus because there are likely fewer studies of such imaging tests for screening in general populations without elevated risk or presentation with symptoms. Nevertheless, it is unclear why the authors would proactively exclude evidence from asymptomatic primary care populations. Similarly, it seems that the review was limited to nonrandomized studies (or were no RCTs identified?). There have been large RCTs of lung cancer screening and test performance could be calculated from the intervention arms. More explanation of the reasoning behind the inclusion criteria for the study population and study design is needed. Justification for the focus on test performance as the main outcome would also strengthen the background and discussion. Did the authors consider any outcomes other than test performance (e.g., stage shift, morbidity, mortality)?

3. The authors imply that the imaging modalities in the review might be considered for screening in primary care populations since these cancers are often discovered at late stages. The populations included in the review (symptomatic) suggest that the results of the review apply to case finding in primary care rather than screening. The point in the limitations section about potential overestimation of test performance relative to what

would be found in an asymptomatic population further imply that the aim of the review is to consider screening in primary care with these imaging tests. More clarity in the discussion with respect to the overall findings is needed, alongside the strength of evidence assessments. What conclusions do the authors drawn with regard the test performance and potential applications to primary care? What level of performance and strength of evidence would be needed?

4. It would be helpful to the reader to include a discussion of the evidence that would be needed to support screening with these imaging modalities in primary care (E.g., levels of test performance, comparative studies with health outcomes, assessment of potential harms). If instead the intent of the review was only to evaluate the test performance of imaging modalities when used as part of a diagnostic pathway when someone presents with symptoms, make this more clear from the beginning.

5. More detail on the approach to narrative synthesis would strengthen the Methods section.

VERSION 1 - AUTHOR RESPONSE

Reviewer: 1<br>Dr. Gilbert Lim, National University of Singapore<br>Comments to the Author:<br>This manuscript is a systematic review evaluating imaging techniques for the early detection of intrathoracic cancers in symptomatic patients in primary care. From over 30,000 candidate papers retrieved from various databases, a relatively small number (13 studies) were included. This resulted in the conclusion that more such studies in primary care settings (with low prevalence) were needed.	We thank the reviewer for their assessment
Some issues might be addressed:<br>1. In the Methods section, the methodology for quality assessment (QUADAS-2) might be described in further detail. In particular, was arbitration with multiple reviewers also done as for screening and data extraction	We have added further detail in the manuscript, see the Methods section:<br>“For quality appraisal, two reviewers assessed the included papers independently, after piloting the approach on the first three papers; disagreements were discussed and consensus was reached. No automation tool was used in the process.”
? The qualifications of the various reviewers (e.g. medical training, years of experience) might also be stated if possible, for all steps in the process.	We have added further detail in the manuscript:<br>While the experience and area of expertise of the reviewers in the team varied, two

	experienced systematic reviewers (BG, JP) ensured the process was methodologically appropriate.
2. In the Methods section, it is claimed that "A number of meetings were organised with patient representatives throughout the project. Patient representatives were invited to provide feedback and suggestions on both the study protocol and the study results, as they became available". Details about these meetings (including any processes such as Delphi consensus), and updates to the protocol/study resulting from such meetings might be stated.	We have revised the Patient and Public Involvement section completely and provided more detail on the stakeholder interaction.<br>"A number of meetings were organised with patient representatives throughout the project. A PPIE panel was established and consulted throughout the project. In the development of the proposal for funding, and in the development of the protocol, the PPIE panel was very supportive of the need for the work and the approaches that we were taking. Two specific issues were raised. First, the suggestion that we should measure regarding test performance in different ethnic groups, led to the potential inclusion of sub-group analysis if the data permitted. The second related to measuring harms as a consequence of testing, as well as issues relating to the process and acceptance to testing (including issues such as waiting times, the need to travel, the need for 'preparation', the stress and anxiety working up to the test). We sought to report on investigate any report of these aspects in the included studies.<br>Patient representatives were invited to provide feedback and suggestions on both the study protocol and the study results, as they became available. At the end of the study we, held a PPIE workshop where we discussed the meaning of the results. The PPIE members were supportive of our findings and conclusions, but again raised issues (beyond

	the scope of the review) about the process and acceptability of testing.”
3. Figure 1 might be rendered as a high-definition image on a single page, instead of being divided across page boundaries as currently.	We have amended the diagram to not be divided across pages.
4. For trial outcomes in Table 3, aggregate statistics such as AUROC/AUPRC might be included, if possible.	As only one of the papers reported relevant AUROC data, the column was eliminated from the table for better page fit. This column has now been reintroduced and a mention was made in the text.
5. Vertical alignment in Appendix 2 might be standardized.	We have amended the supplementary material.
6. NR in Appendix 3 might be clarified.	We have added an explanation in the supplementary material.
7. Some minor spelling/grammatical errors (e.g. "by each reviewe" in the Data Extraction subsection, "An update search" in the Results subsection) might be corrected.	We have revised the entire manuscript and have eliminated all errors identified.
Reviewer: 2<br>Dr. Grace Kim, University of California Los Angeles Jonathan and Karin Fielding School of Public Health<br>Comments to the Author:<br>This is an important study using a systematic search of the literature to identify diagnostic performance of imaging in primary care and studies reporting on impact of diagnostic imaging in primary care, as well as studies reporting the use of artificial intelligence.<br><br>This study includes papers which are relevant for primary care, where participants were suspected intrathoracic cancer and reported on at least one diagnostic performance. The potential bias and the limited of variabilities may be difficulty to rely on the finding of the results for the study question.<br>In the flow chart from Figure 1, 130 papers were assessed in full-text screening. Thirteen papers were included in the final review.	We thank the reviewer for their assessment
Here are major comments<br>(1) Lack of clarification from 130 papers in full-text screening to 13 papers	We have made this clearer in the manuscript.<br>“The most likely reasons for exclusion at full text level wereas the population not being compatible with our inclusion criteria, the non-availability of full text and the lack of reported accuracy data.”

in terms of the detailed methods used to assess risk of bias in the included studies, including details of the tool(s) used, whether they worked independently and the method of training before start review, and, if applicable, details of automation tools used in the process	We have added further detail in the manuscript regarding the risk of bias process.<br>“For quality appraisal, two reviewers assessed the included papers independently, after piloting the approach on the first three papers; disagreements were discussed and consensus was reached. No automation tool was used in the process.”
-what are the major and minor excluded criteria of 117 papers? Although Figure 1 explains the reasons, it was a surprise that there were no imaging studies, and no cancer studies.	The presence of non-imaging / non-cancer studies among the full text papers is due to the fact that, during title and abstract screening, we retained for full text screening entries that either:<br>1. lacked an abstract and/or had an unclear title (e.g. “Analysis of Subcarinal Lymph Nodes in (Suspected) Non-Small-Cell Lung Cancer after a Negative Transbronchial Needle Aspiration – What’s Next?: A Preliminary Report”, DOI: 10.1159/000081765)<br><br>2. title /abstract were to broad to assess details, a side effect of the broad search strategy(ex.” A proposed new imaging pathway for patients with suspected lung cancer”- DOI: 10.1016/j.crad.2011.10.032; “Sonographic evaluation of unexplained pleural exudate: a prospective case series”)
(2) Recommend add forest plot from the meta-study analysis for sensitivity, specificity, PPV, NPV, and possibly accuracy.	As we state in our Methods section, no meta-analysis has been undertaken due to heterogeneity across included studies in imaging techniques, populations, study designs, and outcome measures. However, we have provided now a linked forest plot summarising the sensitivity and specificity reported in each study (Figure

	2). The PPV and NPV estimates are reported in Appendix 3 in the Supplementary material , all with 95% confidence intervals, and estimates of the prevalence of the outcome.
(3) it is uncertain whether two readers are enough to review addressing evidence on the performance of imaging techniques that could aid the detection of intrathoracic cancers in low prevalence settings. When two independent readers disagree (if it happened), is there adjudication or consensus process?	In conducting this review, we followed standard practice of using two independent reviewers (as outlined in the Cochrane methodology, University of York Systematic Reviewing Handbook, and others). We have added further details on the disagreement process in the manuscript:<br>"For quality appraisal, two reviewers assessed the included papers independently, after piloting the approach on the first three papers; disagreements were discussed and consensus was sought. No automation tool was used in the process."
Minor<br>(1) Inconsistent format of references	We have checked and amended the format of references.
(2) In the last paragraph of Discussion, "We identified only one report evaluating the use of an AI system in a primary care context.... This outcome highlights the broader issue of many AI imaging tools, where datasets used for training and validation do not reflect the prevalence of the general population."<br>AI tools can be presented in the multiple papers over time. Do you think that your searching method can cover the results over time? It is quite a surprise that this search found only one paper out of 24,756 screening.	We agree with the reviewer that numbers of papers on the accuracy of AI imaging are ever increasing, and we screened many such papers at the title and abstracts stage. However, given our restrictive inclusion criteria (primary care setting, patients with suspected cancer, accuracy or impact data reported etc.), out of the papers included, only one reported on an AI algorithm.
Reviewer: 3<br>Dr. Robert Ambrosini, University of Rochester Medical Center<br>Comments to the Author:<br>- This paper focuses on the utilization of imaging to detect intrathoracic cancers in primary care settings. The authors conducted a systematic search of the literature to identify	We thank the reviewer for their assessment

studies that looked at imaging including x-ray, CT, MRI, ultrasound, PET, and scintigraphy for detection of intrathoracic malignancy in low prevalence settings.<br>- The paper itself is well-organized and well-written. The Introduction is concise but informative briefly covering each of the included imaging modalities. The Methods section describes the authors approach to the literature search and data processing in satisfactory detail and the Results section includes relatively thorough summaries of each of the relevant papers that were found by the authors during this literature search.<br>- The results of the paper, as stated by the authors, are that only a limited number of relevant studies were available and that there is a need for more studies looking at the value of imaging for detection of intrathoracic malignancy in the primary care setting<br>intrathoracic malignancy detection in the primary care setting, which seems appropriate<br>- As stated by the authors, they are unable to provide any significant synthesis of the results of their literature search due to an overall paucity of relevant papers that were discovered. The Discussion section instead focuses upon the challenges of<br>- The provided Figures/Tables include breakdowns of the authors' search process, which are a positive addition to the manuscript. No medical imaging is provided with this manuscript, however this seems acceptable given the literature search nature of this paper.	
Reviewer: 4<br>Dr. Jillian Henderson, Kaiser Permanent Center for Health Research, Northwest<br>Comments to the Author:<br>The authors present a well-written systematic review of the evidence on different imaging tests used to detect intrathoracic cancers in primary care settings. There are a few areas where the review could be strengthened.<br>1. The study would benefit from the addition of a summary of evidence table that includes certainty ratings for the test performance results with the approach to evaluating certainty outlined in the methods section (E.g., GRADE, EHC<br>https://effectivehealthcare.ahrq.gov/products/methods-guidance-tests-grading/methods).<br>Although the authors describe limitations in the evidence throughout the manuscript, a valuable contribution of systematic reviews is the application of a clear procedure for making determinations about overall certainty.<br>2. The review focuses on studies of the test performance of imaging modalities among symptomatic patients presenting in primary care. Presumably this is the focus	There are a number of points raised by the reviewer that we will respond to together as they are interlinked. These concern the focus of our work around the symptomatic diagnosis of cancers through primary care and issues to do with population screening of asymptomatic people. It seems likely that some of the reviewer's concerns are due to different paradigms, processes and representations of nuanced international differences, especially between the UK and the USA.<br><br>For example, in the UK, there has been increasing focus on the symptomatic diagnosis of cancers through universal primary care, as this is the entry

because there are likely fewer studies of such imaging tests for screening in general populations without elevated risk or presentation with symptoms.<br>Nevertheless, it is unclear why the authors would proactively exclude evidence from asymptomatic primary care populations.	point into diagnostic processes. While CT-screening of high-risk asymptomatic populations is now being implemented in the UK, this would only account for a small percentage of all intrathoracic cancers, with the majority continuing to be diagnosed through a symptomatic route. This is different from the typical pathway in countries such as the USA where insurance coverage provides a faster access to diagnostic imaging – hence more focus on an asymptomatic screening route. These differences are reflected in the international literature.<br><br>This is why our approach to this review was on the symptomatic diagnosis of cancers through universal primary care, and why screening studies of low-risk populations were outside the scope of this review. Furthermore, the test performance of different imaging modalities varies dependent upon the prevalence of the disease in the population under study, and this is markedly different between symptomatic and asymptomatic populations.<br><br>We have made additional clarification of the issues described above in the manuscript, and hope that this provides greater clarity to international audiences.
Similarly, it seems that the review was limited to nonrandomized studies (or were no RCTs identified?).	As specified by the eligibility criteria, all study designs were eligible to be included in the review (including RCTs) as long as they fulfilled the inclusion / exclusion criteria. All outcomes were reported if they were

	available (including stage shift, morbidity, and mortality as the reviewer suggests). Quantitative (accuracy) studies were targeted with the aim of aggregating findings in meta-analyses, even though this was not possible in the end.
There have been large RCTs of lung cancer screening and test performance could be calculated from the intervention arms.	Whilst we agree that test performance could be calculated from the intervention arms in large RCTs of lung cancer screening, we did not set out to do this because the findings would not apply to populations with a different prevalence of disease.<br>As above, this assumption may come from a different paradigm (i.e. asymptomatic screening versus symptomatic presentation in primary care). Screening studies would focus on a different population and we would expect tests to have different performance in this context compared to our population of interest (accuracy metrics are dependent on their place in the clinical pathway, and the level of cancer risk).
More explanation of the reasoning behind the inclusion criteria for the study population and study design is needed. Justification for the focus on test performance as the main outcome would also strengthen the background and discussion. Did the authors consider any outcomes other than test performance (e.g., stage shift, morbidity, mortality)?	This study is part of an initiative to improve cancer detection in primary care. Quantitative (accuracy) studies were targeted with the aim of aggregating findings in meta-analyses, even though this was not possible in the end.
3. The authors imply that the imaging modalities in the review might be considered for screening in primary care populations since these cancers are often discovered at late stages. The populations included in the review (symptomatic) suggest that the results of the review apply to case finding in primary care rather than screening. The point in the limitations section about potential overestimation of test performance relative to what would be found in an asymptomatic population further imply that the aim of the review is to consider screening in primary care with these imaging tests.	We thank the reviewer for the comment relating to our implications that the imaging modalities in the review might be considered for screening in primary care populations since these cancers are often discovered at late stages. However, we contest that we imply this, and having reviewed the manuscript do not propose

	any changes. The focus of the work is on reviewing imaging that might be used for symptomatic patients in primary care. It is possible the reviewer may regard this as 'case-finding'. Our use of the word 'screening' is where testing is applied to asymptomatic populations. As discussed above, and in the manuscript, this is outside the remit of this work – which is why we don't imply this.
More clarity in the discussion with respect to the overall findings is needed, alongside the strength of evidence assessments. What conclusions do the authors drawn with regard the test performance and potential applications to primary care? What level of performance and strength of evidence would be needed?	We have improved the clarity of the findings by including a GRADE assessment and have expanded the discussion to take this assessment into account.
4. It would be helpful to the reader to include a discussion of the evidence that would be needed to support screening with these imaging modalities in primary care (E.g., levels of test performance, comparative studies with health outcomes, assessment of potential harms). If instead the intent of the review was only to evaluate the test performance of imaging modalities when used as part of a diagnostic pathway when someone presents with symptoms, make this more clear from the beginning.	Please see our general response above.
5. More detail on the approach to narrative synthesis would strengthen the Methods section.	We have expanded the Methods section with more detail on the approach and on how we used the extracted data to obtain our results.
